# The Fight for Free Choice of Doctor

**Published:** 1913-02-08

**Authors:** 


### THE FIGHT FOR FREE CHOICE OF DOCTOR.

### Mr. E. B. Turner, F.R.C.S., Describes the London Campaign.

On the front pages of News in The Hospital last Saturday appeared three paragraphs describing the action of a Friendly Society in canvassing on behalf of a panel doctor, which the Insurance officials declared on its immediate challenge by local practitioners to be legal; the difficulties besetting those insured persons who were applying for Form "Med. 21" in their desire to exercise their right to free choice of doctor when the medical man of their choice was not on the panel; and, lastly, the virtual promise of the Government to provide insured persons with the cost of their treatment when they had engaged a doctor off the panel. As it was at once apparent that the great fight for free choice of doctor had come to mean in practice the winning of these three points by insured persons, our representative asked Mr. E. B. Turner, F.R.C.S., who has been one of the chief protagonists of the doctors in their struggle with the Government, to give his views on the situation which has arisen with regard to them.

### The Three Points to Fight for.

"You were quite right," Mr. Turner began, "to juxtapose these three points. The struggle of insured persons for their undoubted right to free choice of doctors is centring upon them; if they insist in sufficient numbers they will win. For it is not the Government, nor the doctors, who hold the key to the position; it is the insured persons who must in the end decide the issue. Take first the question of canvassing. This question is already actively engaging the attention of the British Medical Association. Even before the Act came into force certain panel practitioners began to indulge a propensity for persistent advertising. Street circulars in certain districts—I have one particular offender in my mind—abounded, stating that 'Doctor Panel-ite was ready to treat insured persons and would be happy to call upon them in advance if they desired to make their arrangements for his services.' Such cases the British Medical Association is prosecuting, and the Medical Defence Union is also busy considering how similar offences can be dealt with. Nor will matters stop here. Medical men guilty of persistent advertising have been noted, and will undoubtedly be brought before the General Medical Council at its next session, which will be held in May. There are, of course, abundant precedents for such a course; nor could the Council, on the absurd supposition that it desired to do so, ignore them on the ground that the Insurance Act released panel practitioners from the traditional penalties of professional misconduct. The penalty where such offences have been proved is to be struck off the register, and no Government," Mr. Turner added with ironic humour, "whatever class of practitioners it may employ, can employ those who are not on the register."

### The Doctors' Political Influence.

"We shall hear, you may take it from me, a very great deal more about this. For panel doctors are by no means the only or the chief offenders. As The Hospital noted last week, Friendly Societies are playing the same game, and, apart from the case which you quoted, circulars and broadsides have been pasted in post offices and police stations advertising the claims of imported doctors in districts where the panels have broken down, or 'proved inadequate' is, I think, the current euphemism. As I pointed out in *The Times* some days ago, the importation of doctors into such districts is exactly on a par with the Governmental support of one party in an industrial strike. It is nothing but the introduction of 'blackleg' labour under Government auspices. Were the doctors a trade union no Government would dare by such intervention to risk the loss of trade-union votes, but they think—I say think, for only an election in which the medical profession went out as apostles of the choice of doctor against a party which denied it to the masses could prove the doctor's potential political influence—they think the votes dependent on our good will a negligible quantity. The result is an open practice of strike breaking under Government auspices."

"How about local practitioners' views?"

"Exactly; that is another aspect of the canvassing question. The question is simply this: Are the doctors, whose practices are being injured by the deliberate advertisement of other medical men in their neighbourhood, to be allowed in their turn to advertise with circulars, and to proclaim their merits on public hoardings? The Chancellor of the Exchequer is by no means out of the wood yet. He can foresee as little as anyone how matters will eventuate. The struggle is really only beginning, for the working of the Act has brought more difficulties to its sponsor than any theoretical opposition at an earlier stage."

### The Burden of Clerical Work.

"What is the truth about the clerical work to be performed by doctors on the panel?"

"To start with it may be stated as a principle of efficient treatment that no doctor can adequately look after more than 1,000 patients. They are quite enough for proper attendance and care. Some panel doctors are taking as many as 3,000, and in rural districts where the distances are an important factor fewer numbers present a proportional difficulty. It is, however, a fact that many doctors on the panel have had to sit up till 2 or 3 A.M. signing cards and filling in forms. The cards have to be signed once a quarter, or once a year in any case, and the forms to be filled up whenever, of course, a new patient comes along. Take a concrete case. The doctor sits in his room with the day-book before him, and outside his door there is a long line of persons composed of those who desire to secure his attendance in case of sickness and those who are in immediate need of him. They enter in turn, and are entered also in turn in the day-book. So many are waiting that there is no time to arrange the names in alphabetical order, and certainly not to classify them in any less simple way. Some of these patients are in the same crowd of waiting people on the next or a subsequent morning. On reminding the doctor of this fact he is presented with the problem of turning up his previous notes of the case in the day-book. How is this to be done when the names in the book are in no intelligible sequence? The practical question is: What would happen in an actual epidemic? To give you some idea I may tell you one doctor's experience on the panel. He had taken only 257 patients. Of these, 97 consulted him within five days of medical benefit coming into force. Should he wish to do so, the doctor has no right to limit the number of his insured patients, for by March 31 the remainder of the insured who have not 'chosen' their doctors will be allotted by the Committees equally between the doctors on the local panel."

The Committees and Free Choice.

"Then as to the choice of doctor?"

"The Insurance Committees are definitely trying to stop it. There is no doubt about that. What Dr. Lowry, as quoted by The Hospital last week, said as to the withholding of Form 'Med. 21' is quite true. It is the policy of the fighting section of the British Medical Association and of the London Medical Committee that every insured person who desires it should be accorded his right to this form. The plan of campaign which is being actively organised in London and the suburbs is this: First, to induce insured persons to demand their own doctor, whoever he may be; secondly, to provide the insured persons who desire to make their own arrangements with a requisition for leave to do so—in other words, for Form 'Med. 21.'"

Form "Med. 21."

"Is it being withheld?"

"Orders have evidently been received by local Insurance Committees from headquarters to keep it back. The London and Middlesex Committees are keeping it back. A few other Committees are allowing it to go out. But if there is sufficient demand for it, it must be given. We are doing our best to organise that demand. During the past three weeks I must have addressed 25,000 insured persons in London and the suburbs. These meetings and others have been held in constituencies of every political shade. Districts known to be strongholds of Liberalism and Radicalism, and suburbs of more Conservative tone, have had an equal share of our attention. The result has been surprising. The speeches urging those present to fight for their right to free choice of doctor were received with great enthusiasm. We began to ask ourselves, however, what sort of reception would await us in districts which had returned Radical members at the elections? What would voters say here to our criticism of the working of the Insurance Act with which their members were identified? Instead of interruptions, the speeches were listened to with the tense silence that is the true test of sympathy, and the resolutions, which, by the way, some of our party thought it might be risky to put to the meeting, were carried unanimously. At a meeting held at the Hackney Empire, for instance, at which 4,000 people were present, only ten dissentient hands were held up, and the speakers at this and other meetings were kept very busy at question time. This part of the meeting, instead of wearing the unreality which attends the cheap scores of paid hecklers in the ordinary electioneering campaign, was a true index to the interest felt by the audience, and to their desire to get the best advice that they could."

The Payment of Private Arrangements.

"What is your attitude on the payment of the cost of treatment of those who have made their own arrangements?"

"One Committee has actually suggested that only a penny should be paid by it towards the doctor's bills of those who have contracted out. We tell them that they are entitled to the whole nine shillings. It is true that ambiguity is the mark of this as well as of many other clauses in the benefit arrangements, but if the demand for the whole sum is widely pressed the Insurance Committees will be compelled to show cause why a modification (if any) was intended. Of course, the Government will do all it can to prevent insured persons who have contracted out from getting any sum towards their doctors' bills, and will do everything in its power to feed up the panel practitioners."

"As a matter of fact, they are needing official nourishment badly. For what is actually happening is this: the amount of clerical work, the derogatory and teasing regulations are proving so overwhelming in amount and exasperating in effect that many doctors in London are already anxious to get off the panel. These conditions are leaving them no time to attend to private practice, and the extraordinary situation is resulting that private patients for this reason have therefore left panel doctors to go to men who are not on the panel. A moment's thought shows that this was bound to be the inevitable result of overwork and bad regulations. The panel doctor, therefore, is at his wits' end to know how to deal with insured persons, and the harder he tries to fulfil his duty to them, the more likely is he to lose his private patients. Was there ever such a state of confusion? Were there ever conditions better calculated to exasperate everyone affected by them? The only thing that panel doctors can do, who desire to escape from what has been justly described as 'panel servitude,' is to wait till April 14, and then to decline to renew their contract. That will be the crucial date, when the full extent of the mischief created by the broken pledge will be thoroughly grasped."

Why the Pledge was Broken.

"How do you account for the breaking?"

"It was due to want of co-ordination between the various divisions of the Association. If each of these had realised how few in number the whole-timers were, the members at large would have held out, in spite of the tactics which despatched the same supposititious bands to different districts, and, in fact, falsified the lists. In London, for example, where the profession was well co-ordinated and each division was well informed of the state of affairs elsewhere, the battle was practically won till the meeting was held at which the pledge was abrogated."

"What will be the outcome?"

"No one can say till April 14 indicates the extent of the opposition. That is the crucial date; for by that time insured persons will have begun to realise that they cannot get the doctors whom they like, and that while the panels may comprise a few men of repute, they undoubtedly consist of all the rest who have none. There is a possible chance yet of retrieving the position if the unwilling doctors on the panel, now that they know who are likely to remain, come off in a lump. This is being found out; and its effect would be disastrous to the existing system. If the unwilling doctors decline to renew their contracts on April 14, the panel system must break down, because the men who are willing to remain will have neither the *morale*, the skill, the standing, nor the ability to carry out any system, let alone a bad one."

